# Assessment of Knowledge and Attitudes of Undergraduate Students on Trichomoniasis in Ghana

**DOI:** 10.1002/puh2.70170

**Published:** 2025-12-16

**Authors:** Christopher Yaw Dumevi, Ezekiel Kofi Vicar, Lorie Christla Quansah, Rhoda Oklu, Smith Joshua, Joyce Junior Asiamah, James‐Paul Kretchy, George Boateng Kyei, Patience B. Tetteh‐Quarcoo, Nicholas T. K. D. Dayie, Irene Ayi, Patrick F. Ayeh‐Kumi

**Affiliations:** ^1^ Department of Physician Assistantship Studies School of Medical Sciences Central University Accra Ghana; ^2^ Department of Medical Microbiology University of Ghana Medical School Accra Ghana; ^3^ Department of Clinical Microbiology School of Medicine University for Development Studies Tamale Ghana; ^4^ Department of Public Health School of Medical Sciences Central University Accra Ghana; ^5^ Department of Medicine Washington University School of Medicine St. Louis Missouri USA; ^6^ Department of Virology Noguchi Memorial Institute for Medical Research College of Health Sciences University of Ghana Accra Ghana; ^7^ Department of Parasitology Noguchi Memorial Institute for Medical Research College of Health Sciences University of Ghana Accra Ghana

**Keywords:** attitude, knowledge, reproductive morbidity, sexual behavior, trichomoniasis, undergraduate students

## Abstract

**Background:**

Trichomoniasis is a non‐viral neglected sexually transmitted disease of public health importance. Undergraduate students, often experiencing newfound freedom from family oversight, may engage in risky sexual behaviors, predisposing them to trichomoniasis and other sexually transmitted infections (STIs). This study aimed to assess the knowledge, attitudes, and practices towards trichomoniasis and associated factors among the Central University students in the Greater Accra Region of Ghana.

**Methods:**

An institutional cross‐sectional study was conducted between March and July 2024, involving 387 undergraduate students from Central University who were selected using a simple random sampling technique. Data were collected using a structured researcher‐led interview with respondents in the Schools of Nursing and Midwifery, Pharmacy, and Medical Sciences. The study used bivariate and multivariable logistic regression (STATA MP Version 16) to identify factors linked to trichomoniasis, calculating odds ratios (95% confidence intervals [CI]) to measure associations. Multivariable analysis considered variables with a *p* value <0.05 to be statistically significant factors associated with knowledge and attitudes regarding *Trichomonas vaginalis* infection.

**Results:**

The study included 387 participants (81.40% female, 18.60% male; mean age 20). About 171 (44.19%), 150 (38.76%), and 66 (17.05%) were pharmacy, nursing, and physician assistantship studies students, respectively. Significant associations with *T. vaginalis* infection were observed for gender (*p* = 0.0169), age (*p* < 0.0001), attitude (*p* < 0.0001), and knowledge (68.0% good knowledge; 82.89% good attitude). Females exhibited twice the likelihood of higher knowledge (aOR = 2.022, 95%CI = 1.128–2.912, *p* = 0.012) compared to males.

**Conclusion:**

The study revealed a good level of knowledge, positive attitudes, and effective preventive practices regarding trichomoniasis among the students. Gender, age, program of study, knowledge, and attitudes were significantly associated with self‐reported risk behaviors. The study recommends targeted reproductive health education, particularly on trichomoniasis and STIs, to enhance safer sexual behaviors among tertiary students.

## Graphical abstract

This study assessed the knowledge, attitudes, and practices regarding *Trichomonas vaginalis* among 387 undergraduate students in Ghana. Findings revealed good knowledge (68%) and positive attitudes (82.9%), with gender, age, and academic program significantly associated with awareness and risk behaviors, highlighting the need for targeted reproductive health education.

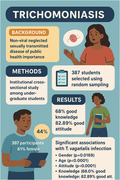

## 1 Introduction

Trichomoniasis is a common, curable, non‐viral sexually transmitted infection (STI) caused by *Trichomonas vaginalis*, a motile extracellular protozoan [1, 2]. Globally, approximately 153 million people are infected, with the highest burden reported in low‐ and middle‐income countries, particularly in sub‐Saharan Africa, where prevalence ranges between 15% and 37% [3]. Females are disproportionately affected, and among sexually active individuals, the prevalence of trichomoniasis exceeds that of gonorrhea, syphilis, and chlamydia combined [4].

Although often asymptomatic, trichomoniasis can cause significant morbidity. In women, symptoms may include dysuria, vaginitis, dyspareunia, and frothy purulent discharge, whereas men may present with urethritis, prostatitis, or epididymitis [5, 6, 7]. Untreated infections are associated with adverse reproductive outcomes, including infertility, preterm birth, low‐birth‐weight infants, and increased risk of cervical neoplasia [8]. Importantly, infection with *T. vaginalis* enhances susceptibility to other STIs, including HIV, HPV, and HSV‐2, thereby amplifying its public health relevance [5, 9].

Young people, particularly university students, represent a high‐risk demographic due to evolving sexual behaviors, increased autonomy, and limited access to accurate sexual health information [10, 11]. Studies from Iran, the United States, and sub‐Saharan Africa indicate inconsistent condom use, multiple sexual partnerships, and limited awareness of STI prevention among individuals aged 15–24 years [12, 13, 14]. These patterns underscore the vulnerability of young adults to trichomoniasis and other STIs, especially when infections are asymptomatic and therefore more easily transmitted.

Despite the significant burden of *T. vaginalis*, data on the knowledge, attitudes, and preventive practices related to trichomoniasis among Ghanaian undergraduates remain scarce. Understanding these factors is critical for designing targeted interventions to improve sexual health literacy and reduce STI transmission. This study therefore aimed to assess the knowledge, attitudes, and preventive practices concerning trichomoniasis among undergraduate health sciences students in Ghana, providing essential insights to inform reproductive health education and public health strategies.

## 2 Methods

### 2.1 Study Design, Setting, and Population

This was an institution‐based cross‐sectional study conducted among undergraduate health sciences students of Central University in Ghana between March and July 2024. A pretested and standardized questionnaire was used to collect data from the study participants. The study was conducted at the main campus of Central University, Miotso, near Dawhenya, in the Greater Accra Region of Ghana, located at 5.5663° N and 0.2410° W. Central University is a co‐educational institution that runs both undergraduate and postgraduate programs. A simple random sampling technique was used to select undergraduate students aged 18 years and above from the Schools of Nursing and Midwifery, Pharmacy, and Medical Sciences, which constituted the study population and the sample size.

### 2.2 Inclusion Criteria

The inclusion criteria were as follows: (a) provision of written informed consent for participation and publication of findings; (b) age of 18 years or older; (c) self‐reported sexual activity; and (d) status as a continuing student at Central University for at least 1 year. Individuals who dissented were excluded.

### 2.3 Ethics Approval

This study was approved by the Institutional Review Board of Central University (CUIRB/10/07/24) and the Ethical and Protocol Review Committee of the College of Health Sciences, University of Ghana (CHS‐Et/M.1‐P 4.2/2023‐2024). Study participants were identified by unique numbers. The personal details of the study participants were kept in locked computer files and secured with passwords to prevent unauthorized access. Data of the study participants were anonymized and unlinked to their details during the data analysis.

### 2.4 Variables, Sample Size, and Sampling

The level of the respondents’ knowledge, attitudes, and preventive practices regarding *T. vaginalis* infection were the dependent variables, whereas socio‐demographic characteristics, such as sex, age, marital status, religion, education, number of children, and number of sex partners, were independent variables. The Cochran's formula—*Z* ^2^ *p*(1 − *p*)/*d* ^2^ [15]—was used to determine the sample size for an unknown population. In the absence of analogous studies within the Greater Accra Region of Ghana, a response distribution of 50% was assumed. The sample size was determined with a 95% confidence level, a 5% margin of error, and a 10% nonresponse rate, culminating in a sample size of 400. Four questionnaires with missing values were excluded from the analysis.

### 2.5 Data Collection Tool

Overall, the questionnaire comprised 53 questions on demographic characteristics, knowledge, attitude, and practice regarding trichomoniasis. The standardized questionnaire was divided into the following sections: (a) socio‐demographic characteristics (10 questions); (b) knowledge of trichomoniasis (23 questions); attitude towards trichomoniasis (9 questions); and preventive practices to mitigate the spread of trichomoniasis (11 questions). The validity of the questionnaire was cross‐checked and authenticated by experts in the field. The questionnaire was pretested on 20 undergraduate students aged between 18 and 30 years as part of a pilot study, but the data generated were not included in the analysis. A standardized questionnaire was designed and modified on the basis of similar studies conducted in Kenya [16] and Ethiopia [17].

### 2.6 Data Analysis

The data were entered into Statistical Package for Social Sciences (SPSS) version 26.0 for Windows 11 (SPSS Inc., Chicago, IL, USA) and subsequently exported into STATA MP Version 16 (STATA Corporation, College Station, USA) for statistical analysis. Descriptive analysis was used to summarize the data in the form of frequencies and percentages. The chi‐square test statistic was then used to determine possible associations between trichomoniasis and the independent variables. Bivariate analyses were conducted to determine the risk factors of trichomoniasis at 95% confidence intervals (CI). A *p* value <0.05 was considered statistically significant.

### 2.7 Assessment of Knowledge, Attitudes, and Sexual Behaviors

#### 2.7.1 Knowledge

Section B of the standardized questionnaire included 23 structured questions designed to assess knowledge on trichomoniasis with 3 possible responses: “yes,” “no,” and “I don't know.” These questions precisely elicited responses and tested the knowledge of respondents on STIs and trichomoniasis. The correct response was awarded 1 point, whereas incorrect response, including “I don't know,” received a score of 0. In this study, if “yes” was the correct answer, a “yes” response received 1 point, whereas “no” or “I don't know” is a score of 0 point or otherwise reverse. When respondents provide 50% or more of the correct answers on the knowledge of trichomoniasis, it is classified as “good knowledge,” and the reverse is classified as “poor knowledge.”

#### 2.7.2 Attitudes

Section C of the standardized questionnaire comprised nine structured questions on the attitude of respondents towards trichomoniasis, with two likely responses: “true” or “false.” This section evaluated the psychological state of respondents concerning their views, opinions, morals, and general dispositions to act in specific ways [18]. Each correct attitude response was awarded 1 point, whereas incorrect responses received 0 points. In this study, if “true” was the correct answer, a “true” response received 1 point, whereas a “false” response received 0 points, and vice versa. When respondents provide 50% or more of the correct answers on the attitudes towards trichomoniasis, it is classified as “good attitude” and the reverse is classified as “poor attitude.”

#### 2.7.3 Sexual Behavior

Section D of the standardized questionnaire evaluated the sexual behavior of respondents towards trichomoniasis. This section had 11 well‐structured questions with three likely responses: “no,” “once in a while,” or “always.” Each correct sexual behavior reported was awarded a score of 1 point, whereas an incorrect option was rated a 0 score. In this study, if “no” was the correct answer, a “no” response received 1 point, whereas “once in a while” or “always” received 0 points, and vice versa. This method of assessment has been used in similar studies [17]. Sectioning of the responses in this manner is the most suitable and convenient approach for studies related to assessing the knowledge, attitudes, and practices (KAP) of respondents regarding trichomoniasis.

Knowledge and attitude questions included a third option, “once in a while” or “I don't know,” to facilitate ease of response by sexually active individuals, particularly those who are undecided or doubtful. The third option always received a 0 score due to the cumulative percentage approach, which only considered the acceptable or correct response. This method converted the total cumulative score to 100%. Therefore, a cumulative score below 70% on KAP was deemed “poor,” whereas a score of 70% or higher was considered “good” [17, 18].

## 3 Results

### 3.1 Socio‐Demographic Characteristics of Participants

Majority of the respondents were females (315, 81.40%) compared to males (72, 18.60%). The mean age of the respondents was 20 years. A total of 368 (95.09%) of the respondents were single, whereas 8 (2.07%) and 6 (1.55%) were married and divorced, respectively. The predominant religion of the respondents was Christianity, 309 (79.84%), followed by Islam, 65 (16.80%), and the African traditional religion, 13 (3.36%). In terms of the program of study, 171 (44.19%) were pharmacy students, whereas 150 (38.76%) and 66 (17.05%) were nursing and physician assistantship studies students, respectively. There were statistically significant associations between participants’ background, such as gender (*p* = 0.0169) and age (*p* < 0.0001), and knowledge about *T. vaginalis* infection (Table 1).

**TABLE 1:** Socio‐demographic characteristics of participants (*n* = 387).

			Good knowledge		Poor knowledge			
Variable	*N*	(%)	*n*	(%)	*n*	(%)	*χ* ^2^	*p* value
Gender								
Male	72	18.60	40	55.56	32	44.44		0.0169
Female	315	81.40	223	70.79	92	29.21		
Age								
16–20	178	45.99	97	54.49	81	45.51	28.80, 2	<0.0001
21–25	153	39.53	120	78.43	33	21.57		
26–30	56	14.47	46	82.14	10	17.86		
Marital status								
Single	368	95.09	252	68.48	116	31.52	0.7516, 2	0.6867
Married	8	2.07	6	75.00	2	25.00		
Divorced	6	1.55	5	83.33	1	16.67		
Religion								
Christianity	309	79.84	215	69.58	94	30.42	1.849, 2	0.3967
Islamic	65	16.80	40	61.54	25	38.46		
African traditional	13	3.36	8	61.54	5	38.46		
Program of study								
BSc Nursing	150	38.76	103	68.67	47	31.33	0.2902, 2	0.8649
BSc Physician Assistantship Studies	66	17.05	43	65.15	23	34.85		
BSc Pharmacy	171	44.19	117	68.42	54	31.58		

### 3.2 Knowledge of STI

**FIGURE 1:**
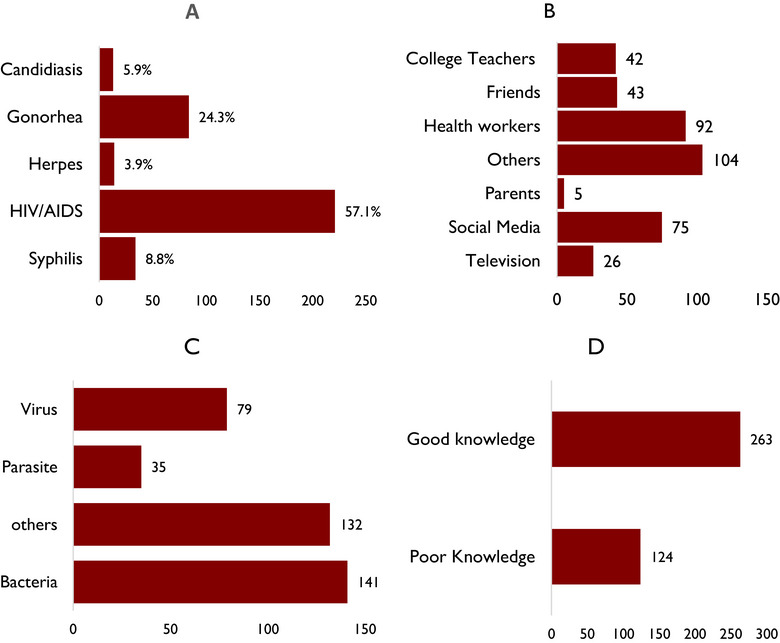
(A) STI known by participants; (B) source of information on *Trichomonas vaginalis*; (C) cause of trichomoniasis; (D) overall knowledge of *T. vaginalis*.

More than half of the respondents, 221 (57.1%), had knowledge about HIV/AIDS, whereas 94 (24.3%), 34 (8.8%), 15 (3.9%), and 23 (5.9%) had knowledge about gonorrhea, syphilis, herpes, and candidiasis, respectively (Figure 1A).

### 3.3 Sources of Information on *T. vaginalis*

In terms of the source of knowledge on *T. vaginalis*, 104 (26.9%) of the respondents got their knowledge from undefine sources, whereas 92 (23.8%), 75 (19.4%), 43 (11.1%), 42 (10.9%), 26 (6.7%), and 5 (1.3%) got their knowledge on *T. vaginalis* from health workers, social media, friends, college teachers, television, and parents, respectively (Figure 1B).

### 3.4 Causative Organism of Trichomoniasis

A total of 141 (36.4%) of the respondents held the view that trichomoniasis was caused by a bacterium, whereas 132 (34.1%), 79 (20.4%), and 35 (9.0%) believed it was caused by an unnamed microorganism, virus, and parasite, respectively (Figure 1C).

### 3.5 Overall Knowledge of *T. vaginalis*

Generally, 263 (68.0%) of the respondents had good knowledge of *T. vaginalis* compared to 124 (32.0%) who had poor knowledge (Figure 1D).

### 3.6 Knowledge of Respondents on *T. vaginalis*

**FIGURE 2:**
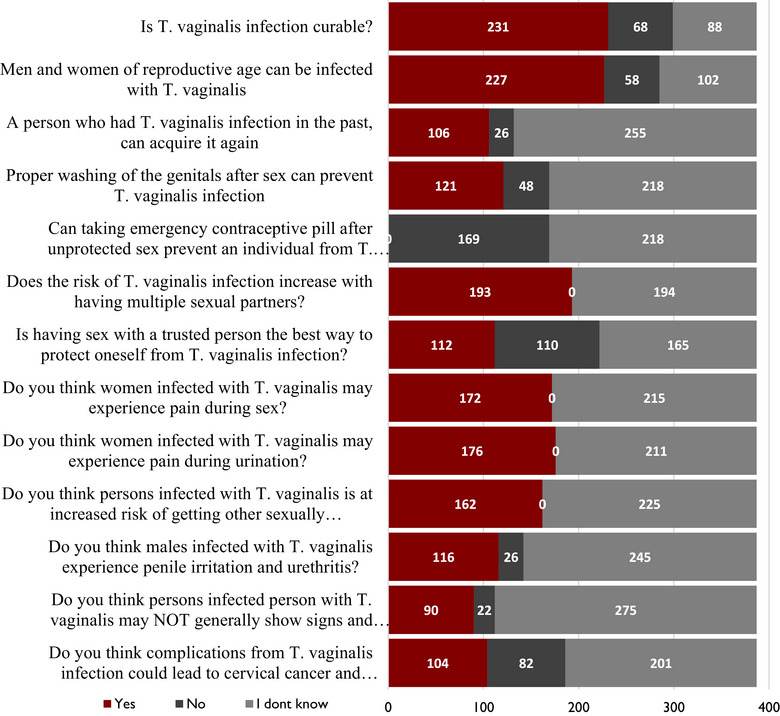
Proportions of responses to knowledge questions answered by participants.

As shown in Figure 2, 231 (60.0%) of the respondents knew trichomoniasis is curable, and 227 (58.7%) believed that men and women of reproductive age can be infected with *T. vaginalis*. About 106 (27.4%) of the respondents knew reinfection of *T. vaginalis* was possible. Although about 121 (%)thought washing the genitals after sexual intercourse can prevent *T. vaginalis* infection, 169 (43.7%) correctly answered that taking emergency contraceptive pills after unprotected sexual intercourse cannot prevent *T. vaginalis* infection. Almost half of the respondents, 193 (49.9%), held the view that having multiple sexual partners increases the risk of *T. vaginalis* infection. Although 112 (28.9%) believed having sexual intercourse with a trusted person was the best preventive measure, 172 (44.4%) and 176 (45.5%) had the view that women with *T. vaginalis* infection may experience pain during sexual intercourse and painful urination, respectively. Additionally, 162 (41.9%), 116 (30.0%), 90 (23.3%), and 104 (26.9%) knew that *T. vaginalis* infection increases the risk of STI/HIV infection, causes penile irritation and urethritis in males, subclinical presentation of symptoms and signs in both males and females, and could lead to cervical cancer and infertility.

### 3.7 Attitude of Respondents Regarding *T. vaginalis*

**FIGURE 3:**
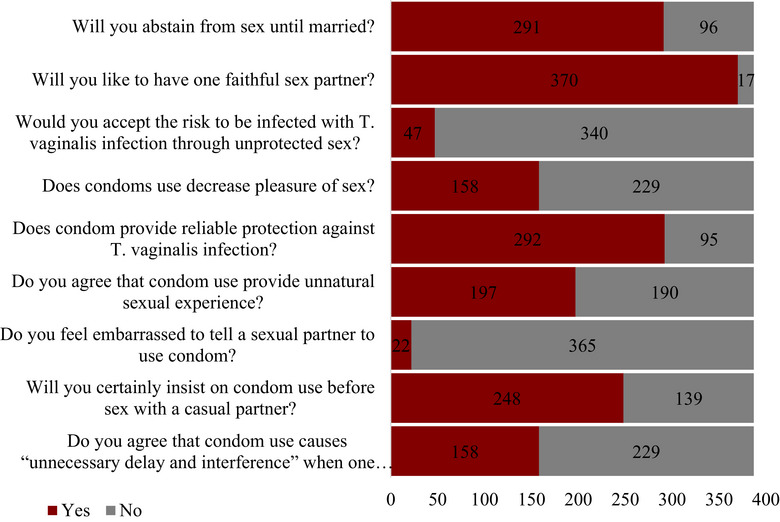
Proportion of responses to questions on attitude by participants.

Of the 387 respondents, 291 (75.2%) agreed to abstain from sex until married. However, 370 (95.6%), 47 (21.1%), 158 (40.8%), and 292 (75.5%) agreed to have one faithful sex partner, were willing to be infected with *T. vaginalis* through unprotected sexual intercourse, and thought condom use decreased the pleasure of sex but was a reliable way of protection against trichomoniasis, respectively. Again, 197 (51.0%) of the respondents believed that condom use provides an unnatural sexual experience, whereas 22 (5.7%), 248 (64.1%), and 158 (40.8%) agreed that they felt embarrassed to tell their sex partners to use condoms, insist on condom use before engaging in sexual intercourse with a casual partner, and that condom use causes unnecessary delay and interference when one is ready for sex, respectively (Figure 3).

### 3.8 Preventive Practices Towards *T. vaginalis*

**FIGURE 4:**
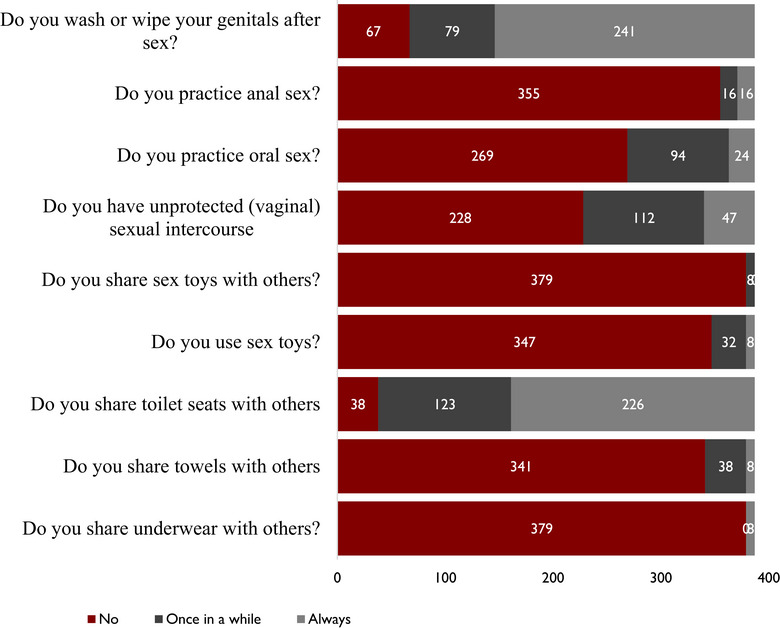
Proportion of responses from all respondents to statements to determine practice of prevention of *Trichomonas vaginalis* infection.

A total of 67 (17.3%) respondents do not wash or wipe the genitals after sex to prevent *T. vaginalis* infection, whereas 355 (91.7%), 269 (69.5%), and 228 (58.9%) do not practice anal sex, oral sex, or unprotected vaginal sex, respectively. Furthermore, 379 (97.9%), 347 (89.7%), 38 (9.8%), 341 (88.1%), and 379 (97.9%) do not share sex toys, use sex toys, share toilet seats, or share towels and underwear, respectively (Figure 4).

### 3.9 Factors Associated With Knowledge of *T. vaginalis*

There were statistically significant associations between participants’ backgrounds, such as gender (*p* = 0.0169), age (*p* < 0.0001), and attitude (*p* < 0.0001), and knowledge about *T. vaginalis* infection. Females were two times more likely to have knowledge of *T. vaginalis* infection than men (aOR = 2.022; 95%CI = 1.128–2.912, *p* value = 0.012). Respondents from the age bracket of 21–25 years (aOR = 2.91; 95%CI = 1.621–5.001; *p* < 0.0001) and 26–30 years (aOR = 4.01; 95%CI = 1.967–8.001; *p* = 0.0012) were three and four times more likely to have good knowledge than those within the age of 15–20 years. Moreover, respondents who had good attitudes were five times more knowledgeable than those with poor attitudes (aOR = 5.03; 95%CI = 4.220–9.121, *p* = 0.0099) (Table 2).

**TABLE 2:** Logistic regression analysis of respondents’ background characteristics and attitude with knowledge of *Trichomonas vaginalis*.

	Good knowledge		Poor knowledge							
Variable	*n*	(%)	*n*	(%)	OR	CI	*p* value	aOR	CI	*p* value
Gender										
Male	40	(55.56)	32	(44.44)	1			1		
Female	223	(70.79)	92	(29.21)	1.939	1.158–3.307	0.0169	2.022	1.128–2.912	0.012
Age										
15–20	97	(54.49)	81	(45.51)	1			1		
21–25	120	(78.43)	33	(21.57)	3.037	1.845–4.935	<0.0001	2.91	1.621–5.001	<0.0001
26–30	46	(82.14)	10	(17.86)	3.841	1.824–7.755	0.0002	4.01	1.967–8.001	0.0012
Attitude										
Poor attitude	74	(46.54)	85	(53.46)	1			1		
Good attitude	189	(82.89)	39	(17.11)	5.567	3.514–8.921	<0.0001	5.034	4.220–9.121	<0.0099

There were statistically significant associations between practice level and the background of respondents, such as gender (*p* = 0.0169), age (*p* < 0.0001), program of study (*p* < 0.0001), attitude (*p* < 0.0001), and knowledge (*p* < 0.0001) regarding *T. vaginalis* infection (Table 3).

**TABLE 3:** Characteristics, attitudes, and knowledge of *Trichomonas vaginalis* versus practice regarding *T. vaginalis* prevention.

		Good practice		Poor practice			
Variable	*N*	*n*	(%)	*n*	(%)	*χ* ^2^	*p* value
Gender							
Male	72	31	(43.06)	41	(56.94)		<0.0001
Female	315	255	(80.95)	60	(19.05)		
Age							
15–20	178	108	(60.67)	70	(39.33)	31.81,2	<0.0001
21–25	153	129	(84.31)	24	(15.69)		
26–30	56	50	(89.29)	6	(10.71)		
Marital status							
Single	368	277	(75.27)	91	(24.73)	2.687, 2	0.2609
Married	8	7	(87.50)	1	(12.50)		
Divorced	6	3	(50.00)	3	(50.00)		
Religion							
Christianity	309	228	(73.79)	81	(26.21)	0.1252, 2	0.9393
Islamic	65	49	(75.38)	16	(24.62)		
African traditional	13	10	(76.92)	3	(23.08)		
Program of study							
BSc Nursing	150	138	(92.00)	12	(8.00)	50.58, 2	<0.0001
BSc Physician Assistantship Studies	66	51	(77.27)	15	(22.73)		
BSc Pharmacy	171	98	(57.31)	73	(42.69)		
Knowledge							
Poor	124	76	(61.29)	48	(38.71)		<0.0001
Good	263	211	(80.23)	52	(19.77)		
Attitude							
Poor attitude	159	98	(61.64)	61	(38.36)		<0.0001
Good attitude	228	189	(82.89)	39	(17.11)		

Referencing Table 4, female respondents were five times more likely to engage in good preventive practices than males (aOR = 5.33; 95%CI = 3.900–10.21; *p* < 0.0001). Respondents in the age range of 21–25 years (aOR = 3.22; 95%CI = 2.335–6.783; *p* < 0.0001) and 26–30 years (aOR = 5.10; 95%CI = 3.32–11.04; *p* < 0.0001) were three and five times more likely to have good preventive practice than those with the age range of 15–20 years.

**TABLE 4:** Logistic regression analysis of respondents’ background characteristics, attitudes, and knowledge with practice regarding the prevention of *Trichomonas vaginalis* infection.

Variable	OR	CI	*p* value	aOR	CI	*p* value
Gender						
Male	1			1		
Female	5.621	3.300–9.625	<0.0001	5.33	3.900–10.21	<0.0001
Age						
15–20	1			1		
21–25	3.484	2.085–5.828	<0.0001	3.22	2.335–6.783	<0.0001
26–30	5.401	2.212–12.44	<0.0001	5.10	3.32–11.04	<0.0001
Program of study						
BSc Nursing	1			1		
BSc Physician Assistantship Studies	0.2957	0.1250–0.6565	0.0026	0.30	0.1200–0.8010	0.003
BSc Pharmacy	0.1167	0.06138–0.2214	<0.0001	0.15	0.0802–0.3321	<0.0001
Knowledge						
Poor	1			1		
Good	2.563	1.608–4.085	<0.0001	3.01	2.208–5.291	<0.0001
Attitude						
Poor attitude	1			1		
Good attitude	3.016	1.871–4.862	<0.0001	3.22	1.991–5.44	<0.0001

In terms of the program of study, students of BSc Physician Assistantship Studies (aOR = 0.30; 95%CI = 0.1200–0.8010; *p* = 0.003) and BSc Pharmacy (aOR = 0.15; 95%CI = 0.0802–0.3321; *p* < 0.0001) were associated with 70% and 85% decrease in odds of preventive practices, respectively, unlike those studying BSc Nursing. Respondents with good knowledge (aOR = 3.01; 95%CI = 2.208–5.291; *p* < 0.0001) were three times more likely to have good preventive practice than those with poor knowledge. Similarly, participants with good attitudes (aOR = 3.22; 95%CI = 1.991–5.44; *p* < 0.0001) were three times more likely to engage in good preventive practice towards *T. vaginalis*.

## 4 Discussion

A total of 387 undergraduate students were surveyed, yielding a high response rate of 99.8%. The findings demonstrate that 68.0% of respondents possessed good knowledge of *T. vaginalis* infection, with comparable knowledge levels observed across nursing, pharmacy, and physician assistantship students. Adequate knowledge of trichomoniasis is critical for enabling early detection, prompt treatment, and effective prevention among young adults.

Our findings are consistent with studies conducted in Ethiopia [17] and Iran [19], which reported relatively high awareness of trichomoniasis. However, the results contrast sharply with reports from Malaysia [20, 21] and São Paulo, Brazil [22], where lower levels of knowledge were documented. These differences may reflect variations in educational curricula, public health priorities, and access to sexual health education across geographic regions.

Notably, despite the high overall knowledge scores, only 9.0% of respondents correctly identified trichomoniasis as a parasitic infection. This gap underscores a key concern: Awareness does not necessarily translate into a strong understanding of the pathogen's biology. The neglect of parasitic STIs within broader sexual health education frameworks may contribute to this finding. Integrating trichomoniasis and other neglected STIs into sexual and reproductive health curricula could enhance students’ diagnostic competence, particularly for those training as future healthcare professionals.

Regarding attitudes, 82.9% of respondents exhibited positive dispositions towards trichomoniasis prevention, with a majority supporting abstinence and monogamy. Although such attitudes align with cultural and religious norms in Ghana, they may not reflect real‐world behaviors, given increasing evidence of inconsistent abstinence among young adults in sub‐Saharan Africa. Misalignment between reported attitudes and actual practices is a known barrier to effective STI prevention [23, 24].

Condom use emerged as a complex determinant of preventive behavior. Although most respondents agreed that condoms provide reliable protection, qualitative findings suggest perceived barriers, including reduced sexual pleasure, social stigma, and inconvenience. These perceptions align with findings from Ethiopia [25] and Nigeria [26], where non‐use of condoms correlated with higher rates of *T. vaginalis* infection. Targeted interventions should therefore emphasize not only the efficacy of condoms but also address misconceptions and socio‐cultural constraints that hinder consistent usage.

Encouragingly, preventive practices were generally strong, with high levels of avoidance of unprotected sex, shared underwear, and sex toys. However, the data revealed occasional engagement in risky practices such as unprotected vaginal intercourse, highlighting opportunities for targeted behavior change communication. Similar to Ethiopian studies reporting 85.8% condom adherence [17], our findings suggest that consistent and structured health education significantly influences safer sexual practices.

This study also highlights important demographic associations. Female students demonstrated better knowledge and preventive practices than males, possibly reflecting heightened health‐seeking behaviors or greater exposure to reproductive health education. Older students (21–30 years) exhibited higher knowledge scores, likely due to greater sexual health experience or increased academic exposure. These findings suggest that interventions targeting younger male students may yield the greatest gains in STI prevention.

Contrary to some reports suggesting potential *T. vaginalis* transmission through shared underwear and sex toys [27, 28], our results indicate minimal engagement in these practices among participants. This may reflect differences in cultural norms or greater awareness of hygiene‐related risks within this study population.

### 4.1 Limitations and Strengths of the Study

#### 4.1.1 Limitations

Although the study provides important insights, several methodological limitations should be considered when interpreting the findings. First, the study was conducted within a single private university and limited to students in health‐related programs, which restricts the generalizability of the results to other institutions and academic disciplines. Second, reliance on self‐reported sexual behavior data introduces potential recall and social desirability biases. Third, although the questionnaire was adapted from established tools and reviewed by experts, it was not subjected to formal psychometric validation, which may affect the precision of some measures.

#### 4.1.2 Strengths

Despite these limitations, the study provides robust baseline evidence on *T. vaginalis* knowledge, attitudes, and preventive practices among future healthcare professionals in Ghana. By identifying specific gaps in awareness and behavioral prevention, the findings support the development of targeted sexual health interventions and inform institutional and national strategies for STI control.

## 5 Conclusion and Recommendation

This study revealed a commendable level of knowledge, positive attitudes, and effective preventive practices regarding *T. vaginalis* infection among undergraduate health sciences students in Ghana. Notably, female students and older students demonstrated significantly higher levels of knowledge and better preventive practices. Despite a high level of knowledge about the protective effects of condoms, a significant proportion of students reported inconsistent condom use, citing reasons such as reduced sexual pleasure. These findings underscore the urgent need for targeted and continuous reproductive health education on university campuses. Such programs should not only focus on the etiology, transmission, and prevention of *T. vaginalis* and other STIs but also address the socio‐behavioral factors that influence sexual health practices. To enhance the generalizability of these findings, future research should be expanded to include multiple universities and a more diverse range of academic disciplines.

## Author Contributions

Christopher Yaw Dumevi, George Boateng Kyei, Patience B. Tetteh‐Quarcoo, Irene Ayi, and Patrick F. Ayeh‐Kumi developed the concept of the study. Lorie Christla Quansah, Rhoda Oklu, and Smith Joshua helped significantly in study participant recruitment and data collection. Ezekiel Kofi Vicar analyzed and interpreted the data. Christopher Yaw Dumevi drafted the manuscript. Joyce Junior Asiamah and James‐Paul Kretchy revised the manuscript. George Boateng Kyei, Patience B. Tetteh‐Quarcoo, Nicholas T. K. D. Dayie, Irene Ayi, and Patrick F. Ayeh‐Kumi supervised the study and significantly contributed to the final draft of the manuscript. The authors read and approve the final manuscript.

## Funding

The authors have nothing to report.

## Ethics Statement

The study was approved by the Institutional Review Board of Central University (CUIRB/10/07/24) and the Ethical and Protocol Review Committee of the College of Health Sciences, University of Ghana (CHS‐Et/M.1‐P 4.2/2023‐2024).

## Conflicts of Interest

The authors declare no conflicts of interest.

## Transparency Statement

The lead author affirms that this manuscript is an honest, accurate and transparent account of the study being reported; that no important aspects of the study have been omitted; and that any discrepancies from the study as planned (and, if relevant, registered) have been explained.

## Data Availability

All relevant data are presented within the manuscript. The dataset used for the analysis can, however, be obtained from the corresponding author upon reasonable request. The corresponding author has full access to all the data in this study and takes complete responsibility for the integrity of the data and the accuracy of the data analysis.
